# Canagliflozin alters the gut, oral, and ocular surface microbiota of patients with type 2 diabetes mellitus

**DOI:** 10.3389/fendo.2023.1256292

**Published:** 2023-10-05

**Authors:** Limin Wang, Chenghong Liang, Xiaojian Song, Xiaoyan Jia, Xiudan Wang, Yun Zhang, Qinyuan Xie, Nan Zheng, Huijuan Yuan

**Affiliations:** ^1^ Department of Endocrinology, Henan Provincial Key Medicine Laboratory of Intestinal Microecology and Diabetes, Henan Provincial People’s Hospital, Zhengzhou, China; ^2^ Department of Endocrinology, Henan Provincial Key Medicine Laboratory of Intestinal Microecology and Diabetes, Zhengzhou University People’s Hospital, Zhengzhou, China; ^3^ Department of Endocrinology, Henan Provincial Key Medicine Laboratory of Intestinal Microecology and Diabetes, Henan University People’s Hospital, Zhengzhou, China

**Keywords:** type 2 diabetes mellitus, canagliflozin, gut microbiota, oral microbiota, ocular surface microbiota

## Abstract

**Background:**

Modifications in the gut microbiota may be a crucial factor in the efficacy of canagliflozin (Cana) in managing patients with type 2 diabetes mellitus (T2DM). However, the interplay between oral and ocular surface microbiota and this treatment remains poorly explored.

**Aim:**

This study aimed to assess alterations in the gut, oral, and ocular surface microbiota pre- and post-Cana treatment in patients with T2DM.

**Methods:**

In this 30-day, controlled before-and-after study, 21 treatment-naïve patients with T2DM received sole treatment with Cana (100 mg/day), and were matched with 10 healthy controls based on gender and age. Using 16S rRNA sequencing, changes in the gut, oral, and ocular surface microbiota pre- and post-Cana treatment were assessed and compared with those of healthy controls. Concurrently, diabetes-related clinical parameters were recorded over the study period. The trial was registered in the Chinese Clinical Trial Registry (ChiCTR200034878).

**Results:**

A noticeable shift was observed in the gut, oral, and ocular surface microbiota pre- and post-Cana treatment. The post-Cana treatment gut microbiota was more similar to that of the healthy controls. Network correlation analysis revealed that modifications in the gut, oral, and ocular surface microbiota were related to changes in clinical parameters, especially for the ocular surface microbiota.

**Clinical parameters:**

A significant decrease in fasting plasma glucose (8.22 ± 2.19 *vs* 6.87 ± 1.09 mmol/L), glycated serum protein [291.00 (264.00, 353.00) *vs* 275.00 (251.00, 342.50) μmol/L], hemoglobin A1c (7.39 ± 1.18 *vs* 7.12 ± 1.33%), body mass index (25.32 ± 2.99 *vs* 24.83 ± 2.95 kg/m^2^), systolic blood pressure (129.05 ± 17.51 *vs* 123.43 ± 14.82 mmHg), and urinary creatinine [158.40 (74.75, 219.15) *vs* 79.70 (56.25, 138.10) μmmol/kg] levels was noted after 30-day Cana monotherapy (*P* < 0.05).

**Gut microbiome:**

Treatment with Cana resulted in an increase in the relative abundance of short-chain fatty acid (SCFA)-producing bacteria, particularly *Lachnospiraceae UCG 004*, *Bacteroides*, and *Lachnospiraceae NK4A136 group*.

**Oral microbiota:**

After Cana treatment, a significant increase of *Prevotella* and *Veillonella*, both of which are known to be closely associated with SCFAs, was observed.

**Ocular surface microbiota:**

Post-Cana administration, the ocular surface microbiota exhibited the most distinct changes in structure and composition. Remarkably, the majority of the increased ocular surface microbiota could produce SCFAs within the gut microbiota.

**Conclusion:**

Cana effectively improved the dysregulated glucose metabolism in patients with T2DM. This improvement can potentially be attributed to the restoration of balance among the gut, oral, and ocular surface microbial communities.

**Clinical trial registration:**

https://www.chictr.org.cn/showproj.html?proj=56487, identifier ChiCTR2000034878.

## 1 Introduction

Canagliflozin (Cana), a sodium-glucose cotransporter 2 inhibitor (SGLT2i), has been used as a therapeutic intervention for type 2 diabetes mellitus (T2DM), serving to reduce glucose reabsorption in the kidneys and augment glucose excretion via urine (1). In addition to ameliorating hyperglycemia, Cana has demonstrated significant beneficial effects on weight management, mitigates cardiovascular disease risk, and relieves diabetic kidney disease (2–4).

In patients with T2DM, gut microbiota imbalance or dysbiosis has been identified, which can potentially aggravate the disease (5). Many studies have shown that the gut microbiota is a pivotal regulator of metabolic disorders, including T2DM, obesity and cardiovascular disease (6–8). In male C57BL/6J mice with diabetic cardiovascular disease, Cana inhibited lipid accumulation, mitochondrial dysfunction by increasing the abundance of short-chain fatty acid (SCFA)-producing gut microbiota, such as *Bacteroides*, *Roseburia*, and *Alloprevotella* (9). Furthermore, a clinical study revealed that empagliflozin, another SGLT2i, can reduce cardiovascular disease risk in patients with T2DM by altering the levels of *Roseburia*, *Eubacterium*, and *Faecalibacterium*, also known as SCFA-producing gut microbiota (10).

As part of the Human Microbiome Project, the oral and ocular microbiomes might exhibit complex and diverse interactions with the gut microbiome, potentially activating inflammatory networks in different organs (11). Both the oral and gut microbiomes are parts of the diverse gastrointestinal environment. Atarashi et al. found that oral pathogens could colonize the gut under conditions of gut microbial dysbiosis, activating intestinal Th1 immunity and leading to chronic inflammation (12). Moreover, A study in germ-free mice have shown that the oral microbiome under T2DM conditions is more pathogenic (13). Whether it can co-contribute with gut microbiome dysbiosis to the onset and progression of T2DM is currently unclear.

Although the ocular surface and the gut are anatomically distant, research on the eye-gut axis suggests that the gut microbiome might participate in the upregulation/downregulation of ocular inflammatory factors via its metabolic products (like SCFAs and lipopolysaccharides) that leak into the systemic circulation, thus altering ocular homeostasis (14, 15). As a mutualistic ocular surface microbiome, it may play a role in local inflammation and immune responses, influencing the onset and progression of T2DM (16). However, how it might interact with the gut microbiome is still largely unknown.

Cana has clear effects on the gut microbiota in T2DM. Although some studies have shown that oral and ocular surface microbiota are associated with T2DM, the effects of Cana on oral and ocular surface microbiota in patients with diabetes remain poorly understood. In this study, we used 16S rRNA gene V3-V4 region sequencing to investigate the changes in the gut, oral, and ocular surface microbiota composition of patients with T2DM pre- and post-Cana treatment, comparing them with healthy controls. The correlation between clinical indicators and the microbiota in each patient was analyzed to explore the possible mechanism of Cana treatment for T2DM. Our results suggest that the regulatory effect of Cana on glucose metabolism may result from a restored balance of gut, oral, and ocular surface microbiota.

## 2 Materials and methods

### 2.1 Clinical study design

This open-label, controlled before-and-after clinical trial was approved by the Ethics Committee of the Henan Provincial People’s Hospital (Eth.2020110) and registered with the Chinese Clinical Trial Registry (ChiCTR200034878). Informed consent was obtained from all participants for the clinical research.

Between December 2020 and September 2021, 21 treatment-naïve patients diagnosed with T2DM were recruited from the Endocrinology Department at Henan Provincial People’s Hospital. They underwent 30 days of Cana (100 mg/d, Janssen-Cilag S.p.A., USA) monotherapy and adhered to a dietitian-designed general diabetes diet with equivalent nutritional content (25% grains and starches, 25% meat, poultry, fish, and eggs, and 50% non-starchy vegetables). The control group consisted of 10 healthy volunteers who had their annual physical examination at Henan Provincial People’s Hospital and were matched with patients with T2DM in terms of age and gender.

Patients were selected based on the following inclusion criteria: (1) aged between 18 and 70 years; (2) treatment-naïve patients diagnosed with T2DM based on the 1999 World Health Organization (WHO) criteria of a fasting plasma glucose (FPG) level of ≥7.0 mmol/L and/or a 2-hour oral glucose tolerance test (OGTT) plasma glucose level of ≥11.1 mmol/L, or those with a self-reported history of T2DM; (3) hemoglobin A1c (HbA1c) levels between 6.5% and 11%; (4) no history of type 1 diabetes mellitus, ocular diseases, immune disorders, or other severe systemic diseases.

Both patients and healthy controls adhered to the following exclusion criteria: (1) usage of hypoglycemic drugs, antibiotics, proton pump inhibitors, corticosteroids, or eye drops in the past 6 months; (2) oral consumption of probiotics or prebiotics in the past 6 months; (3) body mass index (BMI) ≥28 kg/m^2^; (4) wearing of contact or cosmetic lenses; (5) history of gastrointestinal or ocular surgery; (6) intending to conceive, currently pregnant, or breastfeeding; (7) participation in other clinical trials within the past 6 months; (8) history of alcohol abuse or smoking.

### 2.2 Sample collection

Stool, oral, ocular surface, and blood samples from all patients were collected by the same experimenter after 8 hours of overnight fasting at pre- and post-Cana treatment. To ensure the utmost prevention of sample contamination, both participants and experimenters received intensive training in sterile collection techniques for fecal, oral, and ocular surface samples prior to any collection procedures. For fecal samples, we employed a specialized sterile collection kit. Participants were instructed to first void their bladder and subsequently collect a mid-segment fresh fecal specimen. For saliva, participants adhered to a previously established protocol, rinsing their mouth with physiological saline (17). Upon discarding the rinse, we utilized a professional sterile saliva collection kit. As for the ocular surface samples, the surface was anesthetized using 0.5% proparacaine hydrochloride eye drops. A one-time use sterile cotton swab was then used to gather specimens from the subject’s bulbar conjunctiva, which was subsequently stored in a 1.5 mL Eppendorf sterilized tube. To further mitigate the risk of sample contamination, we implemented negative controls during the sampling process. These controls were also sequenced in the experiments to confirm their absence of detectable sequences, thereby verifying the purity of our samples. Blood samples were centrifuged (Multifuge X3R, Thermo Fisher Scientific, USA) at 3000 g for 20 min after standing at 24°C for 30 min to obtain serum. Upon completion of collection or processing, all samples were stored in a -80°C freezer within 5-10 min until use. Healthy controls followed the same method for sample collection.

### 2.3 Clinical indices

Clinical data from the patients were collected twice: at pre- and post-Cana treatment. Meanwhile, data from the healthy controls were obtained at baseline. Demographic and health-related data such as name, gender, age, medication history, surgical history, history of contact lens use, past medical conditions, and dietary habits were collected using comprehensive questionnaires. Height and weight measurements were taken in the morning on an empty stomach, and all participants wore uniform clothing. FPG concentrations were measured using an Automatic Biochemical Analyzer (TBA-120 FR, Toshiba, Japan). Glycated serum protein (GSP) levels were measured using an automatic biochemical analyzer (ADVIA Chemistry XPT, Siemens, USA). Plasma HbAlc concentrations were measured using high-performance liquid chromatography (Bio-Rad D-10, Bio-Rad Laboratories Co., Ltd., Germany). Routine blood tests were performed using the Swelab Alfa Cell analyzer (Boule Diagnostics, AB, Sweden). Urinary microalbumin (UMALB) and creatinine (UCR) levels were assessed using a DCA Vantage Analyzer (Siemens, Chapel Lane Swords Co., Dublin, Ireland). Triglyceride (TG), total cholesterol (TC), high-density lipoprotein (HDL), and low-density lipoprotein (LDL) levels were measured using an automatic biochemical analyzer (Abbott C1600, Illinois, USA).

### 2.4 DNA extraction and 16S rRNA gene sequencing

Stool, oral, and ocular surface samples were processed for DNA extraction and PCR amplification by the same laboratory personnel. Samples were suspended in 790 μL of lysis buffer (comprising 4 M guanidine thiocyanate, 10% N-lauroyl sarcosine, and 5% N-lauroyl sarcosine-0.1 M phosphate buffer at pH 8.0) in a 2 mL tube with 1 g of 0.1 mm glass beads (BioSpec Products, Inc., USA). After vigorous vortexing, they were incubated at 70°C for 1 h and bead-beaten for 10 min. DNA was extracted using The E.Z.N.A.^®^ Stool DNA Kit (Omega Bio-tek, Inc., GA) and stored at -20°C. The V3-V4 region of the 16S rRNA gene was amplified using DNA extracted from each sample as a template. Products from different samples were mixed in equal proportions and sequenced using the Illumina MiSeq platform. Species accumulation curves were plotted to evaluate the adequacy of the sample size and estimate bacterial richness.

### 2.5 Sequencing data analysis

We extracted clean data from raw data using USEARCH (version 11.0.667) based on the following criteria: (1) Sequences from each sample were extracted using their respective index without any mismatch; (2) Sequences with overlaps shorter than 16 bp were eliminated; (3) Overlaps with an error rate exceeding 0.1 were discarded; (4) Post-merge sequences under 400 bp were omitted. After quality filtering, sequences were clustered into unique sets, arranged in decreasing order of abundance to pinpoint representative sequences through UPARSE’s OTU analysis pipeline. OTUs were classified based on 97% sequence similarity to eric, and annotated using the SILVA reference database (SSU138) (18). Alpha and beta diversities were determined based on OTU analysis, with alpha diversity represented by the Shannon, ACE, Chao, and Simpson index. Beta diversity visualized using principal coordinate analysis (PCoA) based on weighted UniFrac distances. The Adonis test was used to analyze the explanatory power of different grouping factors on sample differences, and linear discriminant analysis (LDA) effect size (LEfSe) (lefse 1.1, https://github.com/SegataLab/lefse) was used to identify characteristic microbial populations and interpret inter-group differences (19). Multi-omics correlation analysis of the gut, oral, and ocular surface microbiota and clinical indicators was performed using Spearman’s correlation analysis (Supplementary **Data Sheets 1****-****3** contain all the OTU information). The raw Illumina sequencing information used in this study can be found in the NCBI sequence read archive under accession number PRJNA978958.

### 2.6 Statistical analysis

Data processing and statistical analysis were performed using Excel 2021 and SPSS 26.0 software. Normally and non-normally distributed metric data were presented using mean ± SD and median (interquartile range), respectively. Categorical data are expressed as frequencies or percentages (%). Comparisons between patients with T2DM pre- and post-treatment were made using paired-sample *t*-tests and *Wilcoxon* tests, while comparisons between patients with T2DM and healthy controls were conducted using independent-sample *t*-tests and *Mann-Whitney U* tests. Inter-group comparisons for categorical data were performed using the *Fisher’s* exact test. *P*-value<0.05 was considered statistically significant.

## 3 Results

### 3.1 Cana improves glucose metabolism, weight, and blood pressure

A total of 21 treatment-naïve patients with T2DM were included in this study and received a 30-day monotherapy treatment with Cana, consisting of 12 males and 9 females, with an average age of 51.19 ± 10.69 years, and 10 healthy controls were matched based on gender (5 males and 5 females) and age (51.40 ± 3.06 years). Compared to pre-treatment with Cana, the patients’ glucose metabolism indicators, such as FPG (8.22 ± 2.19 *vs* 6.87 ± 1.09 mmol/L), GSP [291.00 (264.00, 353.00) *vs* 275.00 (251.00, 342.50) μmmol/L], and HbA1c (7.39 ± 1.18 *vs* 7.12 ± 1.33%), as well as systolic blood pressure (SBP) (129.05 ± 17.51 *vs* 123.43 ± 14.82 mmHg), weight (71.36 ± 11.19 *vs* 69.93 ± 10.81 kg), BMI (25.32 ± 2.99 *vs* 24.83 ± 2.95 kg/m^2^), and UCR [158.40 (74.75, 219.15) *vs* 79.70 (56.25, 138.10) μmmol/kg], significantly improved (*P* < 0.05) (**Table 1**).

**Table 1:** Clinical parameters in patients with T2DM pre- and post-Cana treatment and in healthy controls.

Characteristic	patients with T2DM (*n*=21)		healthy controls (*n*=10)	*P1 value*	*P2 value*
	pre-treatment	post-treatment			
Age (years)	51.19 ± 10.69	–	51.40 ± 3.06	–	0.958
Male [n (%)]	12 (57)	–	5 (50)	–	0.503
Weight (kg)	71.36 ± 11.19	69.93 ± 10.81	61.79 ± 7.67	<0.001	0.021
BMI (kg/m^2^)	25.32 ± 2.99	24.83 ± 2.95	23.88 ± 3.05	<0.001	0.224
WHR	0.94 ± 0.07	0.93 ± 0.06	0.86 ± 0.06	0.409	0.005
SBP (mmHg)	129.05 ± 17.51	123.43 ± 14.82	115.50 ± 14.92	0.009	0.044
DBP (mmHg)	78.62 ± 11.56	76.76 ± 12.27	71.30 ± 14.28	0.225	0.137
HR (bpm)	84.24 ± 15.66	79.05 ± 11.55	75.10 ± 5.86	0.057	0.087
FPG (mmol/L)	8.22 ± 2.19	6.87 ± 1.09	4.88 ± 0.43	0.001	<0.001
ACR (mg/g)	21.07 ± 39.16	23.13 ± 39.14	12.42 ± 17.33	0.607	0.512
UMALB (mg)	9.40 (5.00, 15.75)	6.50 (5.10, 19.40)	3.40 (1.88, 6.10)	0.223	0.003
UCR (μmmol/kg)	158.40 (74.75, 219.15)	79.70 (56.25, 138.10)	73.10 (34.45, 93.32)	0.001	0.014
TC (mmol/L)	4.66 ± 0.89	4.79 ± 0.92	4.35 ± 0.75	0.425	0.344
TG (mmol/L)	1.23 (1.02, 1.76)	1.28 (1.06, 1.66)	1.12 (0.74, 1.38)	0.362	0.183
HDL (mmol/L)	1.33 ± 0.36	1.36 ± 0.32	1.32 ± 0.30	0.425	0.982
LDL (mmol/L)	2.43 ± 0.81	2.59 ± 0.79	2.50 ± 0.51	0.275	0.812
GSP (μmmol/L)	291.00 (264.00, 353.00)	275.00 (251.00, 342.50)	–	0.031	–
HbA1c (%)	7.39 ± 1.18	7.12 ± 1.33	5.25 ± 0.32	0.042	<0.001

P1, patients with T2DM pre-treatment vs post-treatment; P2, patients with T2DM pre-treatment vs healthy controls. For data presented as mean ± SD, inter-group comparisons were performed using a paired-sample t-test or independent-sample t-test. For data presented as median (interquartile range), inter-group comparisons were performed using the Wilcoxon test or Mann-Whitney U test. For data presented as percentages, the Fisher’s exact test was used for inter-group comparisons. T2DM, Type 2 diabetes mellitus; Cana, canagliflozin; BMI, body mass index; WHR, hip-to-waist ratio; SBP, systolic blood pressure; DBP, diastolic blood pressure; HR, heart rate; FPG, fasting plasma glucose; ACR, microalbuminuria/creatinine; UMALB, urinary microalbuminuria; UCR, urine creatinine; TC, total cholesterol; TG, triglyceride; HDL, high-density lipoprotein; LDL, low-density lipoprotein; GSP, glycated serum protein; HbA1c, hemoglobin A1c.

### 3.2 Cana alters the gut, oral, and ocular surface microbiota

#### 3.2.1 Cana changes the overall microbiome composition

**Figure 1:**
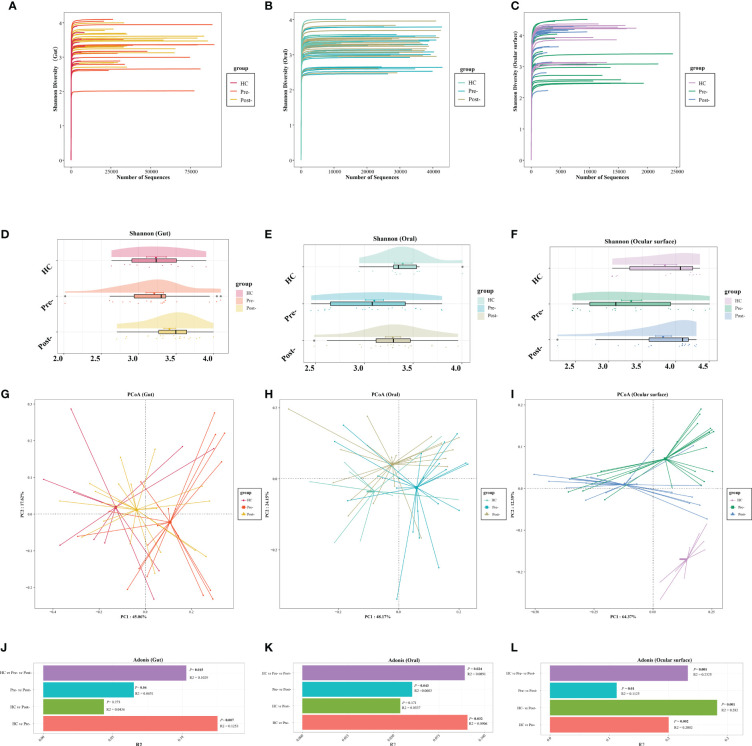
The dilution curves showed that the microbial richness of gut **(A)**, oral **(B)**, and ocular surface **(C)** samples was close to saturation. Across healthy controls and patients with T2DM both pre- and post-Cana treatment, the alpha diversity of the gut **(D)** and oral **(E)** microbiota remained relatively consistent. A noticeable shift was observed in the alpha diversity of the ocular surface microbiota **(F)**. Utilizing weighted UniFrac distances, both PCoA and Adonis analysis revealed marked differentiation in the composition of gut **(G, J)**, oral **(H, K)**, and ocular surface **(I, L)** microbiota pre- and post-Cana treatment in patients with T2DM. Post-Cana treatment, the patients’ gut **(G, J)** and oral **(H, K)** microbiota were more similar to healthy controls. T2DM, type 2 diabetes mellitus; Cana, canagliflozin; PCoA, principal coordinates analysis.

The dilution curve indicated that the sequencing data volume of the microbial samples was judiciously accommodated (**Figures 1A–C**). Compared with pre-Cana treatment, the alpha diversity of the ocular surface microbial communities in patients with T2DM showed an increase in post-Cana treatment (as measured by the Shannon index) (*P*=0.024), while the gut and oral microbiota remained largely unaltered (**Figures 1D–F**, **Supplementary Table 1**). PCoA based on weighted UniFrac distances highlighted a conspicuous segregation trend in the composition of the gut, oral, and ocular surface microbiota following Cana treatment (**Figures 1G–I**). Subsequent Adonis analysis further corroborated these differences, confirming their statistical significance (*P* < 0.05) (**Figures 1J–L**).

To determine if the observed changes were a direct result of drug intervention or influenced by other variables, we incorporated a comparison with healthy controls. Interestingly, when examining beta diversity (as measured by PCoA based on weighted UniFrac), the gut and oral microbial profiles of patients with T2DM post-treatment closely resembled those of the healthy controls (Adonis analysis, *P* > 0.05) (**Figures 1G, H, J, K**). This indicates that Cana treatment may have contributed to restoring a certain balance in the microbiota of patients with T2DM. However, when it comes to the ocular surface microbiota, we didn’t observe this trend. The microbial profiles of the ocular surface in pre-treatment, post-treatment, and healthy controls each displayed unique compositions (**Figures 1I, L**).

#### 3.2.2 Cana impacts the microbiome at various levels

**Figure 2:**
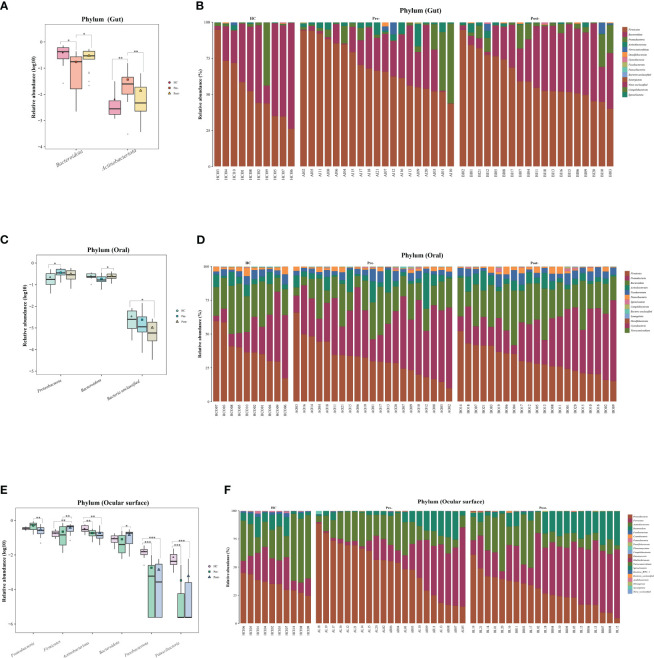
Significant changes in gut, oral, and ocular surface microbiota at the phylum level among healthy controls, patients with T2DM both pre- and post-Cana treatment **(A, C, E)**, and the specific composition of microbiota in each sample **(B, D, F)**. T2DM, type 2 diabetes mellitus; Cana, canagliflozin. Inter-group comparisons were performed using the *Kruskal-Wallis* test. ^*^ *P* < 0.05, ^**^ *P* < 0.01, ^***^ *P* < 0.001.

At the phylum level of the microbial communities, compared to healthy controls, the relative abundance of *Bacteroidota* in patients with T2DM, whether in the gut, oral, and ocular surface, was observed to decrease pre-treatment. However, post-Cana treatment, the relative abundance of *Bacteroidota* in patients with T2DM showed signs of recovery. Moreover, we’ve observed that the microbial compositions of the gut, oral, and ocular surface at other phylum levels also tend to shift closer to that of healthy controls following Cana treatment, especially in the gut microbiota (**Figure 2**).

Based on LEfSe and random forest analyses, we conducted a more detailed exploration of significant variances at both the genus and OTU levels in the gut, oral, and ocular surface microbiota pre- and post-Cana treatment.

**Figure 3:**
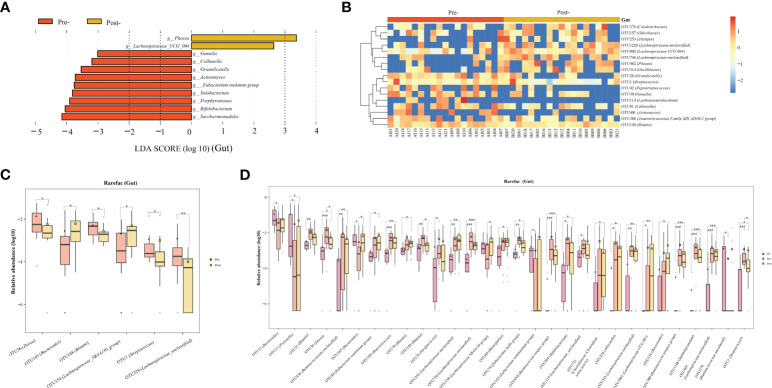
Alterations in gut microbiota of patients with T2DM pre- and post-Cana treatment were evaluated employing LEfSe analysis **(A)** coupled with random forest heatmaps **(B)**. Gut microbiota differences based on the *Wilcoxon* test for OUT level differences in patients with T2DM pre- and post-Cana treatment **(C)**. Gut microbiota with notable OTU level shifts among healthy controls and patients with T2DM pre- and post-Cana treatment (*Kruskal-Wallis* test) **(D)**. T2DM, type 2 diabetes mellitus; Cana, canagliflozin. ^*^ *P* < 0.05, ^**^ *P* < 0.01, ^***^ *P* < 0.001.

Gut microbiota: post-Cana treatment, the relative abundances of *Lachnospiraceae UCG 004* and *Phocea* increased, whereas *Saccharimonadales*, *Bifidobacterium*, *Porphyromonas*, *Solobacterium*, *Eubacterium nodatum group*, *Actinomyces*, *Granulicatella*, *Collinsella*, and *Gemella* diminished (**Figure 3A**). The random forest analysis showed that 17 OTUs, including several divergent genera discerned by LEfSe analysis, demonstrated significant variances pre- and post-Cana treatment (**Figure 3B**). We further performed *Wilcoxon* test on gut microbiota at the OTU levels, finding a significant increase in OTU 165 (*Bacteroides*) and OTU 154 (*Lachnospiraceae NK4A136 group*), and a significant decrease in OTU 36 (*Dorea*), OTU 148 (*Blautia*), OTU 1 (*Streptococcus*), and OTU 329 (*Lachnospiraceae unclassified*) post-Cana treatment (**Figure 3C**). We employed the *Kruskal-Wallis* test to further compare the OTU-level microbiota of healthy controls, patients with T2DM pre-treatment and post-treatment. This further validated that the changes in the microbiota post-treatment were moving in the direction of the healthy controls (**Figure 3D**).

**Figure 4:**
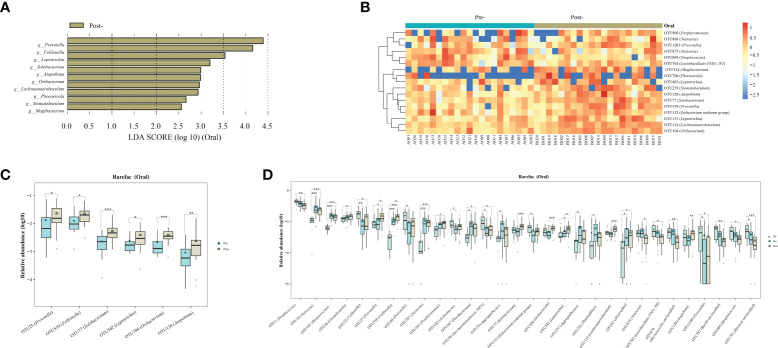
Evaluations of the oral microbiota alterations in patients with T2DM pre- and post-Cana treatment were carried out using LEfSe analysis **(A)** and complemented by random forest heatmaps **(B)**. Oral microbiota with significant OTU level changes in patients with T2DM pre- and post-Cana treatment (*Wilcoxon* test) **(C)**. Oral microbiota with notable OTU level shifts among healthy controls and patients with T2DM pre- and post-Cana treatment (*Kruskal-Wallis* test) **(D)**. T2DM, type 2 diabetes mellitus; Cana, canagliflozin. ^*^ *P* < 0.05, ^**^ *P* < 0.01, ^***^ *P* < 0.001.

Oral microbiota LEfSe analysis revealed a noticeable increase in the levels of *Prevotella*, *Veillonella*, *Leptotrichia*, *Solobacterium*, *Atopobium*, *Oribacterium*, *Lachnoanaerobaculum*, *Phocaeicola*, *Stomatobaculum*, *Mogibacterium*, and *Bacilli RF39* following Cana treatment, which was corroborated by the subsequent random forest analysis and *Wilcoxon* test (**Figures 4A–C**). When we included healthy controls in the three-group comparison of OTU-level microbiota (using the *Kruskal-Wallis* test), we found that OTU 25 (*Prevotella*), OTU 510 (*Veillonella*) and OTU 542 (*Leptotrichia*) that increased post-treatment did not have a relatively high abundance in healthy controls (**Figure 4D**). Interestingly, *Solobacterium*, contrary to its decreasing trend within the gut microbiota, increased in the oral microbiota post-treatment.

**Figure 5:**
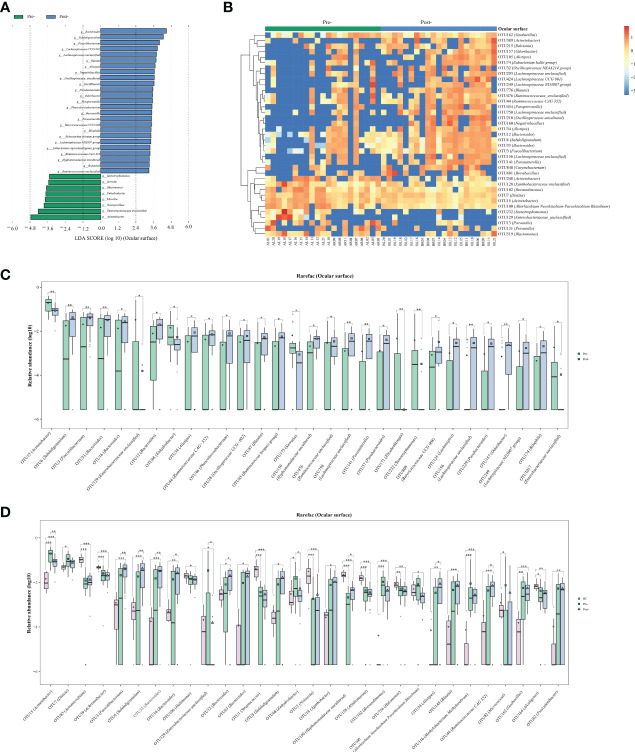
Alterations in the ocular surface microbiota of patients with T2DM pre- and post-Cana treatment were assessed through LEfSe analysis **(A)** and random forest heatmaps **(B)**. Ocular surface microbiota demonstrated significant OTU level changes in patients with T2DM pre- and post-Cana treatment (*Wilcoxon* test) **(C)**. OTU level shifts were identified among healthy controls and patients with T2DM pre- and post-Cana treatment (*Kruskal-Wallis* test) **(D)**. T2DM, type 2 diabetes mellitus; Cana, canagliflozin. ^*^ *P* < 0.05, ^**^ *P* < 0.01, ^***^ *P* < 0.001.

Ocular surface microbiota following Cana treatment, whether compared with healthy controls or before Cana treatment, a notable increase in specific ocular surface microbes such as *Bacteroides*, *Faecalibacterium*, *Lachnospiraceae UCG 001*, *unclassified Lachnospiraceae*, *Blautia*, and *Alistipes*, most of which are known producers of SCFAs in the gut microbiota (**Figure 5**). Concurrently, compared with pre-Cana treatment, a significant decrease in harmful bacteria was noted, such as *Acinetobacter*, which aligned with its lower abundance in healthy controls, along with less-studied ocular surface microbiota, including *Enterobacteriaceae unclassified*, *Enhydrobacter*, *Serratia*, and *Stenotrophomonas* (**Figure 5**). In both the ocular surface and gut, we observed *Bacteroides* with the same rising trend, as well as similar microbiota *Lachnospiraceae UCG 001* and *Lachnospiraceae UCG 004*. However, unlike in the gut, *Blautia* significantly increased on the ocular surface. No ocular surface microbiota exhibiting similarity to the oral microbiomes were identified.

### 3.3 Microbiota-associated clinical benefits of Cana

To elucidate the potential association between microbiome composition and the improved clinical parameters following Cana treatment, we performed Spearman correlation analyses.

**Figure 6:**
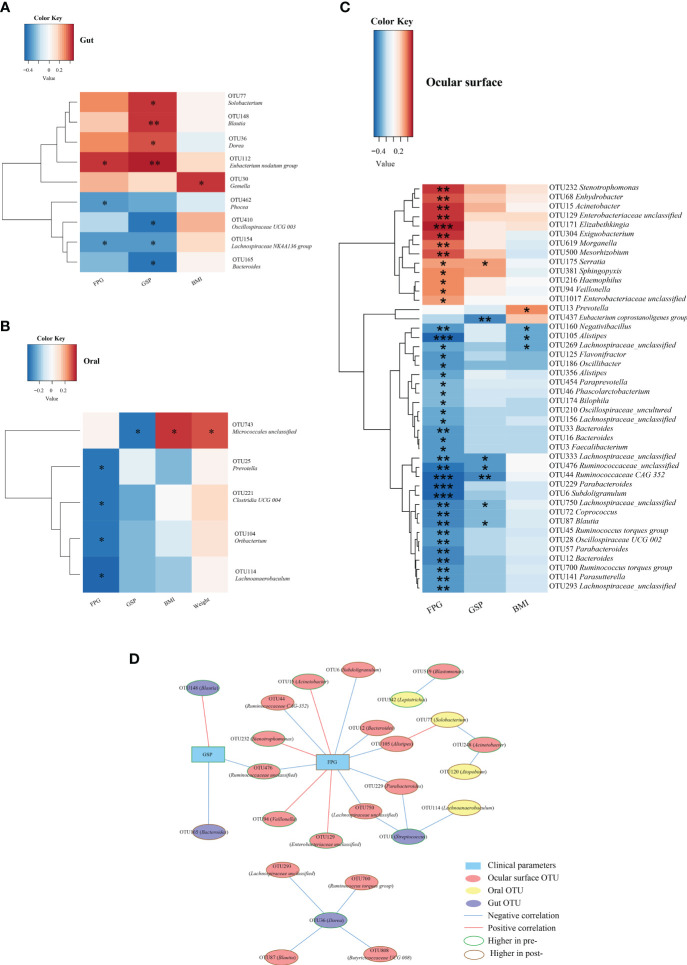
Spearman correlation analysis was conducted to evaluate the interrelation between gut **(A)**, oral **(B)**, and ocular surface **(C)** microbiota and the associated clinical parameters. Network relationship diagram between gut, oral, ocular surface microbiota, and clinical parameters **(D)**. Cana, canagliflozin; FPG, fasting plasma glucose; GSP, glycated serum protein; BMI, body mass index. ^*^ *P* < 0.05, ^**^ *P* < 0.01, ^***^ *P* < 0.001.

Gut microbiota: as illustrated in **Figure 6A**, microbiota such as *Solobacterium*, *Blautia*, and *Dorea*, which decreased post-Cana treatment, showed a significant positive correlation with GPS. In contrast, microbiota such as *Bacteroides* and *Lachnospiraceae NK4A136 group*, which increased post-Cana treatment, exhibited a negative correlation with GSP. Additionally, we also found that FPG negatively correlated with microbiota like *Phocea* and *Lachnospiraceae NK4A136 group*, which increased after treatment, and positively correlated with *Eubacterium nodatum group*, which decreased post-Cana treatment.

Within the oral microbiota augmented post-intervention, *Prevotella* exhibited a negative correlation with FPG (**Figure 6B**). With respect to ocular surface microbiota, the majority of SCFA-producing bacteria that increased following treatment were inversely correlated with glucose metabolism and BMI (**Figure 6C**).

To further investigate the interconnections between the gut, oral, and ocular surface microbiota and clinical indicators, we constructed a network graph demonstrating the alterations pre- and post-Cana treatment (**Figure 6D**). The correlation network diagram suggests that improvements in glucose metabolism indicators were linked to transformations in the gut, oral, and ocular surface microbiota, with a particular emphasis on the ocular surface microbiota.

## 4 Discussion

Our study shows that Cana not only profoundly ameliorates glycemic and cardiovascular metabolism but also yields significant weight reduction in patients with T2DM. Intriguingly, this medication also engenders discernible changes in gut, oral, and ocular microbiota profiles. These transformations potentially represent comprehensive shifts in the mucosal interfaces of patients with T2DM post-Cana administration.

To our knowledge, this is the first comprehensive study to examine the global impact of Cana on the gut, ocular, and oral microbiota in patients with T2DM. Only one animal study has explored the effects of Cana on gut microbiota (9). Notably, we found that *Bacteroides* may play a key role in the Cana treatment of patients with T2DM. Both previous animal study (9) and our clinical research indicate a significant increase in *Bacteroides* after Cana treatment (in the gut), and our research further extends this finding to the ocular surface. Interestingly, while *Bacteroides* shows a high presence in the guts of both healthy controls and patients with T2DM post-Cana treatment, its abundance is low on the ocular surfaces of healthy controls. A clinical study on 98 teenagers revealed *Bacteroides* (7.8%) to be a central component of the ocular surface microbiome in diabetes patients with dry eye syndrome (20). Furthermore, an extensive cohort study examining 10,038 patients with T2DM, who had recently commenced treatment with SGLT2i, indicates that SGLT2i may diminish the onset of dry eye syndrome (21). The protective effect of SGLT2i against this condition might be linked to the presence of *Bacteroides* in the ocular surface microbiota, a hypothesis that certainly merits deeper investigation.

SGLT2i may enhance the metabolic state of patients with T2DM by amplifying the relative abundance of SCFA-producing gut microbiota. Studies involving patients with T2DM treated with empagliflozin and mice with T2DM treated with Cana reported an increase in SCFA-producing bacteria (9, 10). SCFAs have many beneficial effects, stimulating insulin secretion, amplifying insulin sensitivity, and reducing inflammation and oxidative stress (22–24). After Cana administration, in the gut microbiota, we observed an increase in the levels of the SCFA-producing bacterium *Lachnospiraceae UCG 004*, *Bacteroides*, and *Lachnospiraceae NK4A136 group* (25), which showed a negative correlation with GSP, hinting at the crucial role of SCFAs.

The oral-gut microbiota relationship is significant, and we found that Cana treatment might influence oral microbiota via SCFAs, helping blood glucose metabolism. A study investigating oral microbiota and metabolites revealed a positive correlation between salivary SCFA levels and the abundance of the oral bacteria *Prevotella* and *Veillonella*, also known SCFA producers in the gut (26). We observed an increase of *Prevotella* and *Veillonella* in the oral microbiota after treatment, with *Prevotella* negatively correlated with FPG. It’s worth noting that in the healthy population, the relative abundance of *Prevotella* and *Veillonella* is not high. The increased abundance of these two genera might be specific to drug treatment. Interestingly, *Solobacterium*, implicated in colon cancer in gut microbiota (27), increased in the oral but a decrease in the gut post-Cana treatment. The overall effect of *Solobacterium* on patients with T2DM requires further study, considering the potential interactions among the body’s microbiota.

Our study highlights considerable ocular microbiota alterations post-Cana treatment. We discerned a notable increase in the abundance of bacterial genera known to produce SCFAs in the gut microbiota, including *Bacteroides, Lachnospiraceae UCG 001*, *Alistipes*, *Blautia*, *Faecalibacterium*, and others (28, 29). SCFAs (butyrate) generated by gut microbiota can curb ocular inflammation by acting on the SCFAs transporter Slc5a8, which is expressed in the mouse conjunctival and corneal epithelium (14). Based on previous research and the results of our study, we hypothesize that Cana might regulate ocular inflammation through the eye-gut axis mediated by SCFAs. However, this hypothesis warrants further corroboration through metabolomic studies. In addition, based on the network analysis diagram, the improvement of T2DM clinical parameters after Cana treatment might be related to changes in the gut, oral, and ocular surface microbiota, especially the ocular surface microbiota, which provides new insights for the study of the mechanisms of Cana treatment for T2DM.

The strength of our study lies in its direct evaluation of the oral and ocular microbiota in patients with T2DM pre- and post-Cana treatment and the application of network analysis to connect them with the gut microbiota, thereby enhancing data support for eye-gut and oral-gut axis research. Despite its strengths, our study has certain limitations. As it was primarily observational, causality and mechanisms cannot be conclusively established. To deepen our understanding, subsequent metabolomic analyses of saliva and tears in patients with T2DM are warranted, complemented by validation using relevant *in vitro* and animal models. Secondly, while 16S rRNA sequencing serves its purpose, metagenomic sequencing offers greater accuracy. To solidify the findings of this study, future research should lean towards metagenomic sequencing for validation. Lastly, our study was a controlled before-and-after study and was limited by the small number of participants, a short follow-up period. For a more comprehensive insight, upcoming research endeavors should encompass long-term, randomized, double-blind, multicenter cohort studies with age-based subgroup analyses.

## Data availability statement

The datasets presented in this study can be found in online repositories. The names of the repository/repositories and accession number(s) can be found below: https://www.ncbi.nlm.nih.gov/bioproject, PRJNA978958.

## Ethics statement

The studies involving humans were approved by the Ethics Committee of the Henan Provincial People’s Hospital. The studies were conducted in accordance with the local legislation and institutional requirements. The participants provided their written informed consent to participate in this study.

## Author contributions

LW: Writing – original draft, Writing – review & editing. CL: Writing – original draft, Writing – review & editing. XS: Writing – review & editing. XJ: Writing – review & editing. XW: Writing – review & editing. YZ: Writing – review & editing. QX: Writing – review & editing. NZ: Writing – review & editing. HY: Writing – original draft, Writing – review & editing.
