# Supplementary material for: Canagliflozin alters the gut, oral, and ocular surface microbiota of patients with type 2 diabetes mellitus
Source: Front Endocrinol (Lausanne). 2023 Oct 5;14:1256292. doi: 10.3389/fendo.2023.1256292 (PMC10586052; doi:10.3389/fendo.2023.1256292)
Supplement: Supplementary file 4 [file Table_1.docx]

**SUPPLEMENTARY TABLE 1 Statistics of alpha diversity.**

| Microbiota | Shannon index | | | ACE index | | | Chao index | | | Simpson index | | |
| --- | --- | --- | --- | --- | --- | --- | --- | --- | --- | --- | --- | --- |
|  | Pre-treatment | Post-treatment | Healthy controls | Pre-treatment | Post-treatment | Healthy controls | Pre-treatment | Post-treatment | Healthy controls | Pre-treatment | Post-treatment | Healthy controls |
| Gut | 3.30 (2.94, 3.36) | 3.50 (3.27, 3.64) | 3.23 (2.91, 3.51) | 255.71 (222.32, 296.80) | 239.93 (201.72, 307.77) | 240.03 (180.99, 259.48) | 237.06 (217.25, 285.25) | 247.40 (205.86, 312.23) | 239.97 (178.58, 261.21) | 0.09 (0.06, 0.13) | 0.06 (0.06, 0.08) | 0.10 (0.08, 0.12) |
| Oral | 3.08 (2.65, 3.42) | 3.30 (3.12, 3.47) | 3.35 (3.30, 3.53) | 258.94 (235.05, 280.54) | 253.01 (228.82, 269.27) | 247.28 (233.00, 263.72) | 270.04 (231.06, 283.45) | 255.20 (235.05, 276.00) | 261.96 (235.79, 281.08) | 0.10 (0.07, 0.19) | 0.08 (0.07, 0.10) | 0.07 (0.06, 0.09) |
| Ocular surface | 3.11 (2.72, 3.92) | 4.103 (3.60, 4.19) | 4.07 (3.31, 4.25) | 165.86 (126.93, 216.71) | 198.73 (146.11, 223.65) | 259.75 (245.87, 290.13) | 176.60 (108.88, 216.71) | 199.60 (155.25, 225.91) | 288.50 (264.66, 294.55) | 0.08 (0.04, 0.15) | 0.034 (0.03, 0.06) | 0.04 (0.03, 0.11) |
| *P*1 value | 0.254 | | | 0.291 | | | 0.469 | | | 0.164 | | |
| *P*2 value | 0.138 | | | 0.893 | | | 0.837 | | | 0.182 | | |
| *P*3 value | 0.047 | | | 0.003 | | | 0.001 | | | 0.098 | | |

*Data are presented as median (interquartile range), three group comparisons were performed using the Kruskal-Wallis test. P1, gut microbiota; P2, oral microbiota; P3, ocular surface microbiota.*
